# Microbiota composition of fruit flies and their environments in eastern USA orchards

**DOI:** 10.1128/mra.01233-25

**Published:** 2026-01-12

**Authors:** Wendy A. Cisneros Cancino, Joseph T. Gale, Aubrey Cluff, Amanda Morrison, Sarah J. Gottfredson Morgan, Maggie Nosker, Jack K. Beltz, Paul Schmidt, John M. Chaston

**Affiliations:** 1 Department of Plant and Wildlife Sciences, Brigham Young University 6756, Provo, Utah, USA; 2 Department of Biology, University of Pennsylvania 6572, Philadelphia, Pennsylvania, USA; University of Maryland, Baltimore, Maryland, USA

**Keywords:** *Drosophila melanogaster*, east coast, United States, latitude

## Abstract

We present a 16S rRNA analysis of the microbiota of fruit flies and their fruit and soil environments collected across a latitudinal gradient in the eastern USA. Collections varied according to fly taxonomy, location, sampling substrate, and starvation condition. These samples reveal variation in microbiota composition with several key variables.

## ANNOUNCEMENT

To understand how fruit fly microbiota composition is related to latitude, we collected and analyzed the microbiota in flies and environmental samples across a latitudinal gradient in the eastern USA. Many samples are reported in a companion manuscript that focuses on *Drosophila melanogaster* (1). Here, we report a snapshot of microbiota variation across fly taxonomic boundaries from samplings taken at the same time.

In Fall 2021, we collected flies from eight orchards in the eastern USA (Table S10 in reference 1). We sampled flies from individual apples or compost, and cores of adjacent apples flesh (1/4″ × 1/2″), and soil (1/2″ × 3/4″). Samples were immediately stored on dry ice or, for some flies, starved for more than 2 hours at ambient temperatures, then stored long-term at −80°C. Individual male flies were examined by microscopy to make taxonomic assignments. *D. simulans* was discriminated from *D. melanogaster* by the presence of a distinct genital arch. *D. suzukii* was identified based on wing melanization patterns, and *Zaprionus* spp. by the presence of longitudinal black and white stripes. We also found several samples of a black fruit fly species, possibly *D. hydei*, based on its dark color and relatively large body size. Individual flies were transferred to 96-well homogenization tubes, and DNA was extracted using the Zymo Quick-DNA Fecal/Soil Microbe 96 Kit (D6011). 16S rRNA V4 marker gene sequencing was performed using a dual-barcoding approach (2). PCR reactions were normalized, pooled (96 samples/pool) using the Just-a-Plate Normalization Kit (Charm Biotech, JN-120-10), and concentrated (Zymo gDNA Clean & Concentrator 11-302C). Fragments ranging from 250 to 450 bp were selected on a Sage Science Blue Pippin, pooled in equimolar volumes, and sequenced with other samples on two Illumina MiSeq runs using 500 cycle v2 chemistry. The relevant, including samples in reference 1, samples represented 10.1 million demultiplexed reads that passed default Illumina quality filters. At each position, all but four first quartile quality scores were >30.

**Fig 1:**
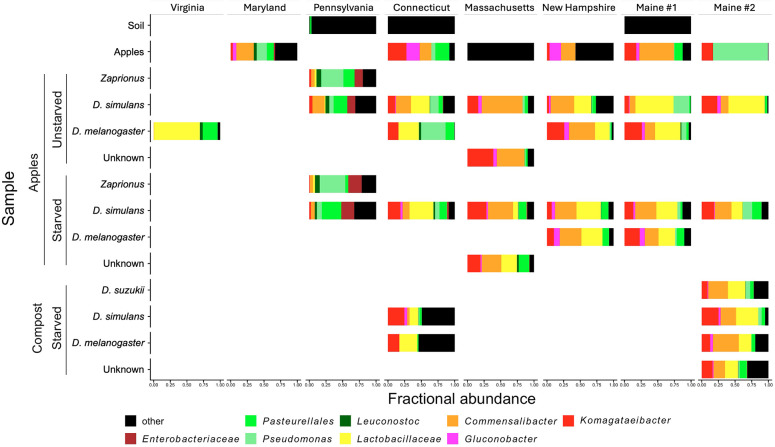
Microbiota composition of wild flies and their environments. Reads were rarefied to 153 reads per sample, clustered at the genus level, and aggregated by the metadata. Genera <2% overall abundance are clustered in the “other” category.

Sequences were analyzed using QIIME2 2022.11 (1, 3–10) as in our previous work. Default parameters were used except where noted. After removing the 291 samples reported previously, we retained 2,452,972 reads in 162 samples (Fig. 1). Reads assigned to *Wolbachia* were removed to analyze the non-reproductive tract microbiota of the flies. Bray-Curtis distances varied with sample type (soil, apples, and flies), sampling location, substrate (apples or compost), fly species, *Wolbachia* presence (present if >25% of total reads), and starvation condition. ANCOM identified bacterial genera that varied between conditions (Table 1) (11). These samples provide additional insight into diet-dependent and geography-dependent variation in microbiota composition of wild *Drosophila* and *Zaprionus* species.

**TABLE 1:** PERMANOVA and ANCOM analysis

	df	SS	*R* ^2^	*F*	*P*	Genera that differ by ANCOM
All samples						
Sample type (soil, fruit, flies)	2	2.66	0.04	4.34	<0.001	*Commensalibacter*
Location	7	11.94	0.17	5.56	<0.001	*Acetobacter*, *Commensalibacter*, *Dysgonomonas*, *Gluconobacter, Komagataeibacter*, *Pseudomonas*, Un. Bacteria, Un. Pasteurellales
Species	4	2.81	0.04	2.29	<0.001	*Acetobacter*, *Commensalibacter, Corynebacterium*, *Dysgonomonas*, *Gluconobacter*, *Komagataeibacter*, *Leuconostoc,* Un. Bacteria, Un. Pasteurellales, Un. Xanthomonadaceae*, Weissella*
Apples or compost	1	1.39	0.02	4.53	<0.001	*Gluconobacter*, *Leuconostoc*, Un. Pasteurellales, Un. Xanthomonadaceae, *Weissella*
Residual	167	51.18	0.73			
Total	181	69.98	1			
Fly samples						
Location	6	12.08	0.2	6.79	<0.001	*Acetobacter*, *Commensalibacter*, *Dysgonomonas*, *Gluconobacter, Komagataeibacter*, Un. Xanthomonadaceae, Un. Pasteurellales,
Species	4	2.71	0.04	2.29	<0.001	*Acetobacter*, *Corynebacterium*, *Dysgonomonas*, *Gluconobacter, Komagataeibacter*, *Leuconostoc,* Un. Pasteurellales, Un. Xanthomonadaceae
Apples or compost	1	1.33	0.02	4.49	<0.001	Un. Pasteurellales
Starvation condition	1	0.23	0	0.76	0.73	Un. Pasteurellales
*Wolbachia* presence	1	0.58	0.01	1.97	0.02	*Dysgonomonas*
Residual	148	43.56	0.72			
Total	161	60.48	1			

## Data Availability

Sequences were deposited in the Sequence Read Archive under BioProject accession PRJNA1306182 and BioSample accessions SAMN51281715:SAMN51281944. Metadata is available at https://github.com/johnchaston/Gale2024/raw/refs/heads/main/MRA_EastCoastExtra_metadata.xlsx.
